# Plant Growth-Promoting Endophytic Bacterial Community Inhabiting the Leaves of *Pulicaria incisa* (Lam.) DC Inherent to Arid Regions

**DOI:** 10.3390/plants10010076

**Published:** 2021-01-01

**Authors:** Amr Fouda, Ahmed M. Eid, Albaraa Elsaied, Ehab F. El-Belely, Mohammed G. Barghoth, Ehab Azab, Adil A. Gobouri, Saad El-Din Hassan

**Affiliations:** 1 Department of Botany and Microbiology, Faculty of Science, Al-Azhar University, Nasr City, Cairo 11884, Egypt; aeidmicrobiology@azhar.edu.eg (A.M.E.); albraa.mahmoud@azhar.edu.eg (A.E.); elbelely@azhar.edu.eg (E.F.E.-B.); mohamed_gamal.sci@azhar.edu.eg (M.G.B.); or saad_hassan@azhar.edu.eg (S.E.-D.H.); 2 Department of Biotechnology, College of Science, Taif University, P.O. Box 11099, Taif 21944, Saudi Arabia; e.azab@tu.edu.sa; 3 Botany and Microbiology Department, Faculty of Science, Zagazig University, Zagazig 44519, Sharkia, Egypt; 4 Department of Chemistry, College of Science, Taif University, P.O. Box 11099, Taif 21944, Saudi Arabia; a.gobouri@tu.edu.sa

**Keywords:** *Pulicaria incisa*, bacterial endophytes, plant growth-promoting, IAA production, antagonistic activity, *Zea mays*

## Abstract

In this study, 15 bacterial endophytes linked with the leaves of the native medicinal plant *Pulicaria incisa* were isolated and identified as *Agrobacterium fabrum*, *Acinetobacter radioresistant*, *Brevibacillus brevis*, *Bacillus cereus*, *Bacillus subtilis*, *Paenibacillus barengoltzii*, and *Burkholderia cepacia.* These isolates exhibited variant tolerances to salt stress and showed high efficacy in indole-3-acetic acid (IAA) production in the absence/presence of tryptophan. The maximum productivity of IAA was recorded for *B. cereus* BI-8 and *B. subtilis* BI-10 with values of 117 ± 6 and 108 ± 4.6 μg mL^−1^, respectively, in the presence of 5 mg mL^−1^ tryptophan after 10 days. These two isolates had a high potential in phosphate solubilization and ammonia production, and they showed enzymatic activities for amylase, protease, xylanase, cellulase, chitinase, and catalase. In vitro antagonistic investigation showed their high efficacy against the three phytopathogens *Fusarium oxysporum*, *Alternaria alternata*, and *Pythium ultimum*, with inhibition percentages ranging from 20% ± 0.2% to 52.6% ± 0.2% (*p* ≤ 0.05). Therefore, these two endophytic bacteria were used as bio-inoculants for maize seeds, and the results showed that bacterial inoculations significantly increased the root length as well as the fresh and dry weights of the roots compared to the control plants. The *Zea mays* plant inoculated with the two endophytic strains BI-8 and BI-10 significantly improved (*p* ≤ 0.05) the growth performance as well as the nutrient uptake compared with an un-inoculated plant.

## 1. Introduction

Agricultural activities in the 20th century were largely accomplished using agricultural equipment, high-yielding crop varieties, intensive tillage, irrigation, fertilizers, pesticides, and other manufactured inputs [1]. These applications can negatively affect the quality of the soil and contribute to environmental pollution [2]. To reduce these negative impacts due to conventional farming techniques, microbial inoculations such as biofertilizers are gaining more attention. A symbiotic linkage is formed between microorganisms and plants, which is useful for both partners. The coexistence of the microbe in the plant affects the health and growth performance of the plant, which effectively improves the agricultural properties, such as the root length and the fresh and dry weight of the shoot and root, and improves the yield productions, quality of the soils, and the nutrient cycling [3,4,5].

Endophytes are a group of symbiotic microorganisms vastly spread through all plants, and they colonize the intracellular and intercellular spaces of the host plant without causing any considerable morphological modification or visional infection [6]. The endospheric microbes produce secondary active metabolites that protect plants from phytopathogens, in addition to exo-enzyme production, which could be aiding plant colonization. Endophytes may enhance plant growth by phytohormone production and support plant growth under adverse biotic and abiotic stress [7]. Recently, bacterial endophytes have been used in different biotechnological sectors, such as bio-fertilizers to improve crop production and significantly reduce the chemical input into the environment [8,9], as well as in nanotechnology for the fabrication of various nanoparticles incorporated in different applications [10,11,12].

Plant growth-promoting endophytes (PGPE) inhabit various plant tissues and form a useful relationship that facilitates the nutrient exchange and improves other activities [13,14]. Phytohormone production by endophytes is one of the best-studied mechanisms of plant growth promotion, leading to morphological and architectural changes in plant hosts [15]. Endophytic microbes possess the pivotal ability to solubilize insoluble phosphate and to supply nitrogen to their host plants [16,17]. Endophytes colonize various plant tissues without any symptomatic disease and, thus, compete with phytopathogens for the same environmental niches. Therefore, endophytes improve plant growth performance and plant health through various mechanisms and may contribute to protecting the plants against phyto-pathogenesis [18].

Endophytic microbes produce many bioactive compounds that have different biological activities that can be plant growth-promoting (PGP) directly or indirectly. Most plants harbor endophytes within their tissue; however, the information available on PGPE and its biological activities is not proportional to the high distribution of endophytes. A superior understanding of the native microbial endophytes in plants may clarify their capabilities and their potential in promoting plant growth and creating a sustainable system for crop production [19].

The genus *Pulicaria* belongs the family *Asteraceae*, which is found in North Africa, among other places. This plant contains a group of bioactive compounds, including alkaloids, tannins, flavonoids, and phenolics that are used in medicinal applications, such as anticarcinogen, antiinflammation, antioxidant, antibacterial, and antiaging agents, in addition to cardiovascular protection and the prevention of many chronic diseases [20]. *Pulicaria* is used by the native populations of Upper Egypt and by the Bedouins as a decoction and tea substitute, as well as in the treatment of heart diseases. The endophytic microbes associated with *Pulicaria incisa* have not yet been investigated, even though this plant is characterized by its therapeutic, biological, and ecological importance. To date, we have found no published study concerning the isolation, identification, and characterization of the endophytic bacteria associated with *P. incisa*.

The current study proposes that the endophytic bacteria associated with *P. incisa* plants are inoculants with potential PGP properties. Therefore, in this study, we mainly aimed to isolate, identify, and characterize the supposed endophytic bacteria associated with *P. incisa,* which inhabits the naturally arid conditions of the Saint Katherine Protectorate. Plant growth-promoting traits of these endophytic bacterial isolates, such as enzyme production, indole-3-acetic acid (IAA) and ammonia production, phosphate solubilization, and antagonistic activity against phytopathogenic fungi were evaluated to determine their impact on the growth performance of maize plants as an important economic crop.

## 2. Results

### 2.1. Isolation and Identification of Bacterial Endophytes

In this study, 15 bacterial isolates, encoded PI-1 to PI-15, were obtained from healthy leaves of *Pulicaria incisa* collected from Wadi al-Zwatin, Saint Katherine, South Sinai, Egypt. These bacterial endophytes underwent morphological, physiological, and biochemical identification according to standard keys. The data showed that the obtained bacterial isolates belonged to two different phyla, namely, proteobacteria (33.3%) and firmicutes (66.6%).

The proteobacterial strains belonged to α, γ, and β and were identified as *Agrobacterium fabrum* (two strains coded PI-1 and PI-2), *Acinetobacter radioresistant* (two strains coded PI-3 and PI-4), and *Burkholderia cepacia* (one strain coded PI-15), respectively, while the firmicutes strains were related to *Brevibacillus brevis* (three strains coded PI-5 to PI-7), *Bacillus cereus* (two strains coded PI-8 and PI-9), *Bacillus subtilis* (two strains coded PI-10 and PI-11), and *Paenibacillus barengoltzii* (three strains coded PI-12 to PI-14). According to these identifications, we selected one strain from each species to confirm their identification using 16S rRNA (16S ribosomal RNA) gene fragments sequence analysis.

**Figure 1:**
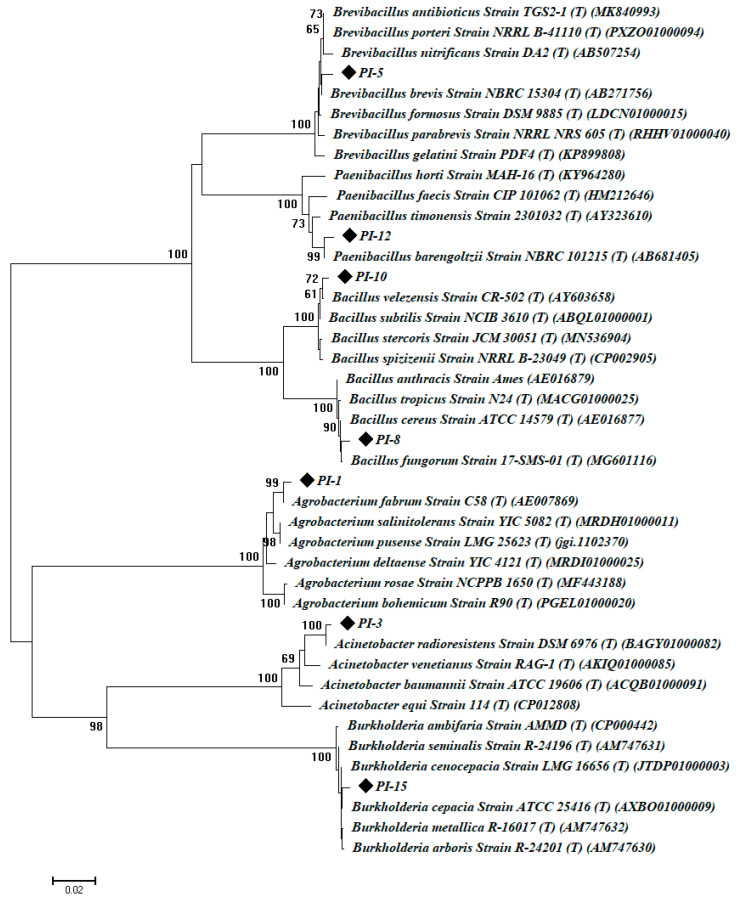
Phylogenetic analysis of 16S rRNA sequences of the bacterial isolates with the sequences retrieved from NCBI (National Center for Biotechnology Information). The analysis was conducted with MEGA 6.1 using the neighbor-joining method with bootstrap value (1000 replicates).

Therefore, seven endophytic bacterial strains, coded PI-1, PI-3, PI-5, PI-8, PI-10, PI-12, and PI-15, were identified based on amplification and sequencing of the 16S rRNA gene. These isolates were identified as *Agrobacterium fabrum* PI-1, *Acinetobacter radioresistant* PI-3, *Brevibacillus brevis* PI-5, *Bacillus cereus* PI-8, *Bacillus subtilis* PI-10, *Paenibacillus barengoltzii* PI-12, and *Burkholderia cepacia* PI-15. The BLAST (Basic Local Alignment Search Tool) analysis of the identified bacterial species showed a percent similarity of 99% compared with the 16S rRNA-related sequence in GenBank (Table 1, Figure 1).

### 2.2. Characterization of Endophytic Bacterial Strains

#### 2.2.1. Salt Tolerance

The identified endophytic strains exhibited varied salt tolerance and their growth was reduced with increasing NaCl concentration in growth media. As seen in Table 1, γ-negative bacteria, *A. radioresistant* PI-3, and *B. cepacia* PI-15 were more sensitive to NaCl and showed weak growth at 150 mM NaCl. By increasing the salt concentration, their growth was inhibited. α-Proteobacteria (*A. fabrum)* and firmicutes species (*B. brevis, B. cereus, B. subtilis,* and *P. barengoltzii)* exhibited high salt tolerance up to 200 mM, and their growth was inhibited by increasing the NaCl concentration above this level.

#### 2.2.2. Indole Acetic Acid (IAA) Synthesis

**Figure 2:**
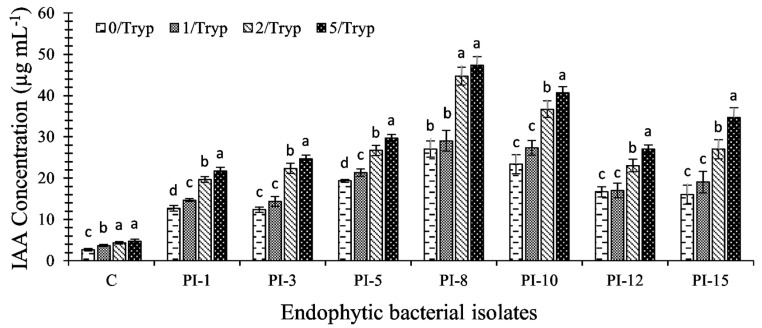
Quantitative assay for indole-3-acetic acid (IAA) production by endophytic bacterial strains at 0, 1, 2, and 5 mg mL^−1^ tryptophan after 14 days. C denotes the control, which was growth media without bacterial inoculation. Identity of endophytic bacterial strains (PI-1: PI-15) is shown in Table 1. Data are statistically different at *p* ≤ 0.05, (*n* = 3); error bars are means ± SE (Standard Error). For each strain, bars with different letters denote that mean values are significantly different at a significance level of *p* ≤ 0.05.

Seven bacterial endophytes, namely, PI-1, PI-3, PI-5, PI-8, PI-10, PI-12, and PI-15, were quantitatively screened to produce IAA after 14 days in the growth medium with the absence and presence of 1.0, 2.0, and 5.0 mg mL^−1^ tryptophan as a precursor for IAA. The results, as presented in Figure 2, showed that all tested bacterial endophytes possessed the efficacy of synthesis of IAA with or without tryptophan. In the absence of tryptophan, the productivity of IAA significantly differed between the different bacterial endophytes and ranged from 12 ± 0.7 to 23 ± 2 μg mL^−1^.

The bacterial strains *Brevibacillus brevis* PI-5 and *Bacillus subtilis* PI-10 showed the maximum IAA production (*p* ≤ 0.001), recording 19 ± 0.3 and 23 ± 2 μg mL^−1^, respectively. However, the addition of L-tryptophan at different concentrations to the growth media significantly increased the IAA production (*p* ≤ 0.001). Analysis of variance showed that, by increasing the tryptophan concentration from 1.0 mg mL^−1^ to 5.0 mg mL^−1^, the IAA productivity increased from 14 ± 1.0 – 27 ± 1.7 to 21.7 ± 0.8 – 47 ± 2.0 µg mL^−1^. The endophytic bacterial isolates *Bacillus cereus* PI-8 and *Bacillus subtilis* PI-10 exhibited the highest IAA production in media amended with tryptophan and produced 47 ± 2.0 and 40.7 ± 1.6 µg mL^−1^, respectively, after 14 days.

**Figure 3:**
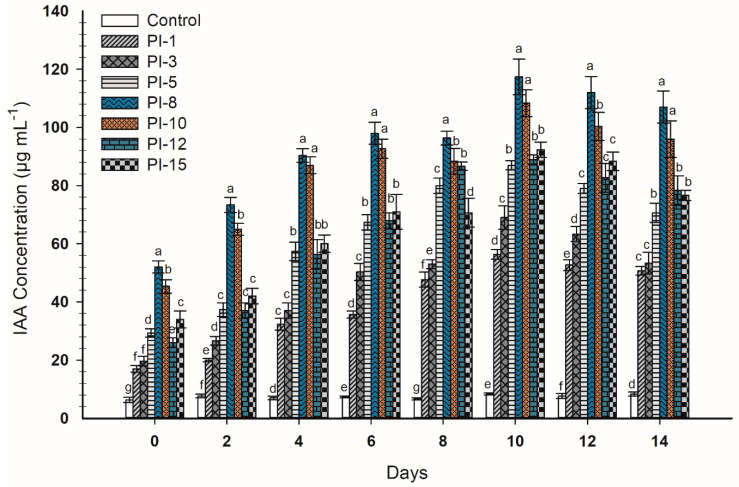
Quantitative production of IAA by endophytic bacterial strains in the presence of 5 mg mL^−1^ tryptophan and at different interval time courses. Control denotes growth media supplemented with 5 mg mL^−1^ without bacterial inoculations. Bars with different letters denote that mean values are significantly different at a significance level of *p* ≤ 0.05 (*n* = 3).

According to the results above, 5 mg mL^−1^ was selected as the best tryptophan concentration to investigate the efficacy of bacterial endophytes to produce IAA at different time courses (2–14 days) (Figure 3). Data analysis highlighted that the highest productivity of IAA was achieved on the 10th day for all isolates with varied amounts ranging from 56 ± 1.7 to 117 ± 6.0 µg mL^−1^. The endophytic bacterial strains PI-8 and PI-10 had the highest IAA production (117 ± 6.0 and 108 ± 4.6 µg mL^−1^, respectively) at day 10 (*p* ≤ 0.05) (Figure 3).

#### 2.2.3. Extracellular Lytic Enzymatic Activities of Bacterial Endophytes

Data showed that all endophytic strains were able to produce xylanase, cellulase, and catalase, while only four strains were positive for the amylase enzyme. In the same regard, six bacterial strains were positive for protease and five were positive for the chitinase enzyme. Notably, the highest amylase, protease, xylanase, and chitinase production were assigned to the endophytic bacterial strain *Bacillus cereus* PI-8 with clear zones of 12.8 ± 0.2, 17.5 ± 0.3, 16 ± 0.1, and 15 ± 0.2 mm, respectively, followed by the bacterial strains *Bacillus subtilis* PI-10 and *Paenibacillus barengoltzii* PI-12. Analysis of variance showed that the endophytic strains PI-8 and PI-10 still possessed the highest enzymatic activities (*p* ≤ 0.001) compared with other endophytic strains (Table 2).

#### 2.2.4. Ammonia Production and Solubilization of Phosphate

The obtained results highlighted the potency of isolated bacterial endophytes for ammonia production. This potentiality varied between low to high ammonia production based on the color change of the inoculated growth media after adding Nessler’s reagent. The analysis showed that four endophytic bacterial strains, identified as *Agrobacterium fabrum* PI-1, *Acinetobacter radioresistant* PI-3, *Bacillus cereus* PI-8, and *Bacillus subtilis* PI-10, could solubilize inorganic phosphate, forming clear zones ranging between 5.6 ± 0.4 and 9.8 ± 0.8 mm. Endophytic strains of PI-8 and PI-10 had the highest efficacy in ammonia production and phosphate-solubilizing activity (Table 3).

#### 2.2.5. In Vitro Antagonistic Activity

**Figure 4:**
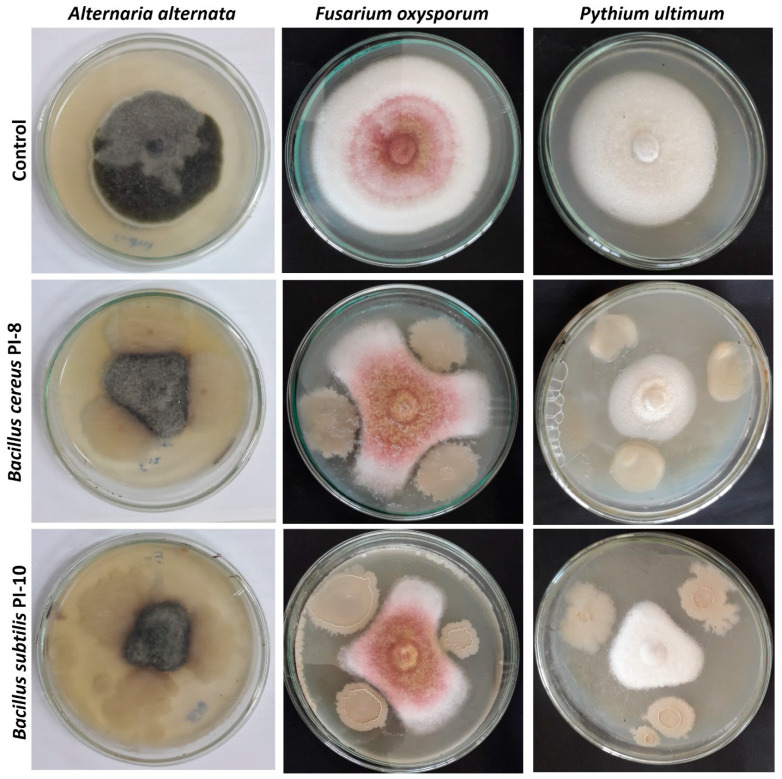
In vitro antagonistic activity of *Bacillus cereus* PI-8 and *Bacillus subtilis* PI-10 against three phytopathogenic fungi of *Fusarium oxysporum, Alternaria alternata,* and *Pythium ultimum* as compared with the control.

The data represented in Table 4 show the in vitro antagonistic activities of isolated bacterial endophytes against three phytopathogenic fungi, represented by *Fusarium oxysporum, Alternaria alternata,* and *Pythium ultimum.* Data analysis showed that the average inhibition percentages of fungal growth were 35.8%, 35.7%, and 18.9% for *F. oxysporum, A. alternata,* and *P. ultimum,* respectively. The highest inhibition percentages were accomplished due to treatment with endophytic bacterial strains of *Bacillus subtilis* PI-10 and *Bacillus cereus* PI-8 (Table 4, Figure 4). The bacterial isolate *Bacillus subtilis* PI-10 had a high capacity to inhibit fungal growth with percentages of 52.6%, 50.07%, and 24.4% for *F. oxysporum, A. alternata,* and *P. ultimum,* respectively, as compared with those recorded by *Bacillus cereus* PI-8 (48.9%, 46%, and 20%, respectively). *Acinetobacter radioresistant* PI-3 exhibited the lowest activity against *A. alternata,* and *P. ultimum,* with recorded inhibition percentages of 22.5% and 15.9%, respectively. The bacterial isolate *Agrobacterium fabrum* PI-1 exhibited the lowest activity against the phytopathogen *F. oxysporum*, with an inhibition percent of 22.3%.

### 2.3. Bio-Inoculations of Maize Root by the Most Potent Bacterial Strains

**Figure 5:**
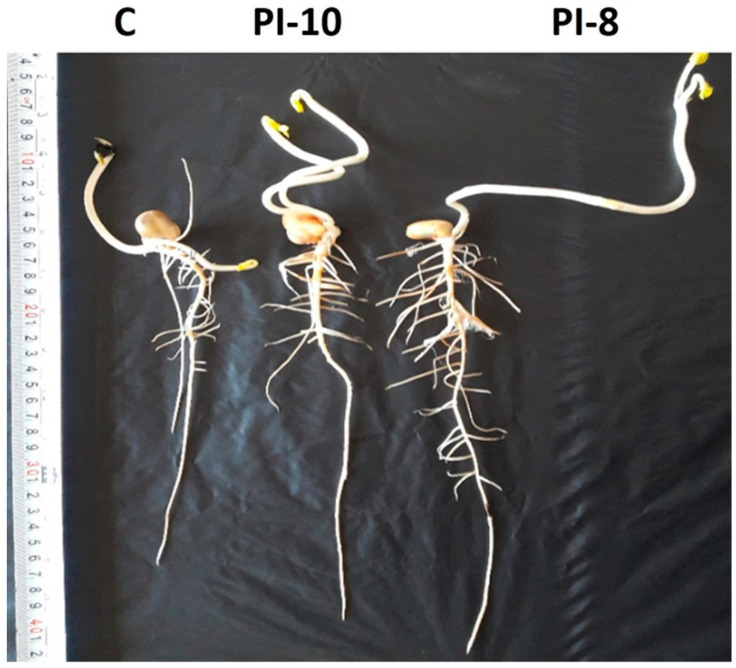
Effect of the two most potent bacterial endophytes as bioinoculant on root growth of representative maize seedlings.

The efficacy of endophytic bacteria as bio-inoculants to plants was assessed using two representative bacterial strains, PI-8 and PI-10, which were selected according to the previous PGP activities. Analysis of variance indicated that bacterial inoculation significantly (*p* ≤ 0.001) increased the root length compared to the controls (not inoculated seeds). Inoculation with PI-8 resulted in longer maize roots compared to PI-10 inoculation. Regarding the biomass weight of inoculated maize roots, multi-comparison analyses showed the bacterial inoculation significantly improved the fresh as well as the dry weight of treated plants compared to the control (*p* ≤ 0.001). However, both inoculants resulted in the promotion of fresh weight with similar values. Maize roots inoculated with PI-8 had higher recorded dry weights compared with those inoculated with PI-10 (Table 5, Figure 5).

### 2.4. Greenhouse Experiment

**Figure 6:**
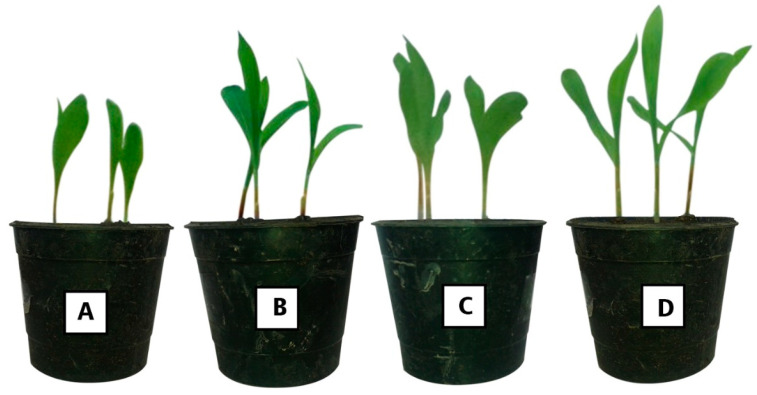
Greenhouse experiment showed the effect of two most potent endophytic bacterial isolates and their consortium on growth performance of *Zea mays* L. plant. (**A**) Control; (**B**) *Zea mays* seeds inoculated with *Bacillus cereus* PI-8; (**C**) *Zea mays* seeds inoculated with *Bacillus subtilis* PI-10; (**D**) *Zea mays* seeds inoculated by bacterial consortium.

The heights of the *Zea mays* plants, due to endophytic bacterial inoculations, were significantly increased (*p* ≤ 0.001) compared to the un-inoculated plants. The height due to *Bacillus cereus* PI-8 inoculation (38.9 ± 0.3 cm) was better than those recorded for *Bacillus subtilis* PI-10 (34 ± 0.8 cm), while the consortium (44.9 ± 0.2 cm) was better than individual strains (Figure 6, Table 6). The growth performance, including the fresh and dry weights for shoot and roots, differed significantly differed (*p* ≤ 0.05) compared with the un-inoculated plants (Table 6).

Data analysis showed that maize plant inoculated with the consortium of two endophytic bacterial strains (PI-8 and PI-10) produced shoot and root fresh weights (718 ± 3.5 and 1918 ± 5.5 mg, respectively) that significantly increased compared with the controls and with plants inoculated with individual strains. The dry weight of the shoots and roots of maize plants inoculated with the bacterial consortium were higher than those recorded for either the control or individual strain (Table 6). Analysis of variance showed that the P shoots of plants inoculated with the bacterial consortium (0.34 ± 0.01%) were significant compared with other treatments (Table 6). On the other hand, the percentages (%) of K and N due to endophytic treatments were not significant compared with the controls.

## 3. Discussion

*Pulicaria incisa* is a common plant in Egypt’s desert inhabits throughout the Sahara, including the Red Sea region and Sinai. It thrives in non-saline wadi beds and has a broad ecological latitude from sandy to gravelly soils with a preferable habitat in sandy–loamy soils. It is characterized by an aromatic smell and various essential oil contents. *P. incisa* has been reported to be used in Sudanese traditional medicine as an antispasmodic, hypoglycemic, tonic, and as an ingredient of perfume [20]. *P. incisa* is also consumed by Egyptian Bedouins instead of common tea [21,22]. This plant contains different physiological and biological components that allow it to thrive in arid regions, which is of value and importance, and part of its diverse adaptation may depend on its ability to establish a viable relationship with microbial endophytes [23].

In the current study, 15 bacterial endophytes were obtained from the surface-sterilized healthy leaves of the medicinal plant *P. incisa.* These bacteria are related to two major phyla, proteobacteria represented by 33.3% of the total isolates and firmicutes, represented by 66.6%. In the same regard, Costa et al. [24] showed that the endophytic strains obtained from leaves of *Phaseolus vulgaris* belonged to proteobacteria, firmicutes, and actinobacteria with a percentage of 36.7%, 32.9%, and 29.7% of the total bacterial isolates, respectively.

To confirm identifications, we selected seven endophytic bacterial isolates belonging to different species for molecular identification. Genotypic identification of the selected seven endophytic isolates showed the obtained strains were related to different genera of *Agrobacterium*, *Acinetobacter, Brevibacillus, Bacillus, Paenibacillus,* and *Burkholderia*. The predominance of the *Bacillus* genus as bacterial endophytes has been isolated from the medicinal plants *Fagonia mollis* and *Achillea fragrantissima* [8], ginseng (*Panax ginseng* C.A. Meyer) [25], *Lonicera japonica* [26], and soybean (*Glycine max* L.) [27]. Hanna et al. [28] also reported the frequency of genus *Bacillus* in several perennial and annual plants found in the north Sinai deserts in Egypt.

*Bacillus* is the most common bacterial species identified as endophytes [29,30]. The obtained results are compatible with those reported that found *Bacillus* spp. widely distributed as endophytic strains in various therapeutic plants, such as *Glycyrrhiza* spp., *Pinellia ternata*, *Digitalis purpurae*, *Lycium chinense*, *Leonurus heterophyllus*, *Belamcanda chinensis*, *Bletilla striata*, *Pinellia pedatisecta*, *Taxus yunnanensis, Fagonia mollis,* and *Achillea fragrantissima* [8,31,32,33].

*Acinetobacter radiresistens* was previously isolated as an endophytic strain from *Phaseolus vulgaris* leaves [24]. *Agrobacterium* and *Burkholderia* were identified as culturable endophytic bacterial strains isolated from soybean plants [34]. On the basis of our online survey, this is the first report for isolation and identification of *Agrobacterium fabrum* from medicinal plant as endophytic strain. To our knowledge, this is the first report regarding the isolation and identification of the supposed bacterial endophytes associated with healthy leaves of *P. incisa,* as well as characterization of these endophytes for used in the future for plant growth promotion.

Wide screening techniques include culture-based and non-specific methods for determining the association and understanding the relationship between plants and endophytes. In this direction, the characterization of plant-related endophytes would be beneficial for biotechnological applications of these microbes in different sectors, such as biofertilizers. Microbial endophytes enhance plant growth either directly or indirectly through various mechanisms, such as the synthesis of plant hormones, particularly IAA, phosphorus solubilization, salt tolerance, or abiotic stress amelioration, producing ammonia or hydrolytic enzymes as well as inhibiting phytopathogenic microbes [6,7,35]. Therefore, in this study, we evaluated the traits for bacterial endophytes that could be described as plant growth-promoting.

All bacterial isolates had the efficacy to grow in up to 200 mM salt stress, except for *Acinetobacter radiresistens* and *Burkholderia* *cepacia,* which could be grown up to 150 mM NaCl. The phenomenon of salt stress can help plants that are sensitive to saltiness if these bacterial endophytes are used as bio-inoculants. The potentiality of these endophytic bacteria to ameliorate salt stress can be attributed to several strategies including osmoprotectant accumulation in their cytoplasm.

Osmoprotectants are compounds that do not carry any charge at physiological pH, have high solubility, and are non-toxic in large concentrations. In addition to the osmoprotectants, many compatible solutes can be consumed as a nitrogen and carbon source for bacteria [36] or form exo-polysaccharides that bind cations including sodium, thus impeding the sodium absorption by plants [37]. The indirect mechanism is through the synthesis of auxin, which enhances the root elongation under the influence of salinity to provide nutrients to the plant. Thus, indole-producing bacteria participate in the plant’s adaptation to salt as was observed in date palm trees [38].

Endophytic microbes that produce IAA have a significant role in mutualistic interactions between the host plant and endophytes and, hence, regulate the plant growth [39]. Data analysis showed that the efficacy of endophytic bacteria in producing high IAA concentrations was significantly improved with increasing tryptophan levels. L-tryptophan is a physiological precursor to auxin production in microorganisms and higher plants.

Furthermore, the application of exogenous L-tryptophan at low concentrations manifested a positive effect on growth parameters of maize plants, while increasing concentration to 25 mg kg^−1^ soil negatively affects the shoot dry weight and leaf width [40]. Interestingly, all isolated bacterial endophytes had the capacity to synthesis IAA in culture media in the absence of tryptophan, which expresses the normal conditions inside the plant tissues, where IAA synthesis is achieved in the absence of tryptophan levels.

In this study, the highest IAA concentration values of 117.3 and 108.3 µg mL^−1^ were assigned for bacterial endophytes of *Bacillus cereus* PI-8 and *Bacillus subtilis* PI-10, respectively, after 10 days of incubation in the presence of 5 mg mL^−1^ tryptophan, which produced positive indicators for these endophytes to enhance root development and promote plant growth. As compared with other studies, the obtained endophytic bacterial strains recorded highest IAA production. Hassan [19] reported that *Bacillus cereus* and *Bacillus subtilis* isolated from shoot system of *Teucrium polium* L. collected from the same area have the ability to produce IAA with highest values ranging between 4.1.0 and 23.4 µg mL^−1^ in the presence of 5 mg mL^−1^ tryptophan. The obtained data in this study were compatible with the result of AlKahtani et al. [8], who showed that the highest IAA production using bacterial endophytes associated with the two medicinal plants, *Fagonia mollis* and *Achillea fragrantissima*, was attained in the presence of 5 mg mL^−1^ tryptophan after 10 days of incubation.

The root growth improvements are considered the main phenotypic characteristics for IAA production [41]. In the same regard, the leaf endophytic *Sphingomonas* sp. obtained from *Tephrosia apollinea* produced gibberellins and IAA, and tomato plants inoculated with this endophyte showed enhanced growth traits (dry weights of root and shoots, the chlorophyll content, and shoot lengths) [42]. IAA has a crucial role in plant root formation and cell division stimulation, even under harsh environmental conditions [41,43].

Our results supposed that the bacterial endophytes obtained in this study potentially regulate plant growth through IAA synthesis. Different species of endophytic microbes can produce variable levels of IAA in a way that differentially affects plant growth. Previously, Dobbelaere et al. [44] observed that the IAA synthesized by various bacterial endophytes enhanced plant growth and, hence, promoted growth of the root area, which subsequently improved the nutrient uptake from the soil. Dawwam et al. [45] showed that bacterial endophytes synthesized various amounts of IAA ranging from 0.36 mg mL^−1^ to 14.77 mg mL^−1^ and from 0.6 mg mL^−1^ to 10.73 mg mL^−1^. Two endophytic fungi, *Penicillium chrysogenum* and *Alternaria alternata*, and non-spore-forming fungus were isolated from the medicinal plant *Asclepias sinaica* and were found to exhibit high IAA productions ranging between 100 and 160 mg mL^−1^ and displayed a significant increase in the root length of maize plants [46].

The production of different hydrolytic enzymes by endophytic microbes indirectly promotes plant growth and protects from phytopathogens [47]. In this study, all tested bacterial endophytes obtained from *P. incisa* produced xylanase, cellulase, and catalase, while four bacterial isolates (57% of tested strains) produced amylase, six bacterial strains (85.7% of tested strains) were found to be potent protease producers, and five bacterial isolates (71% of tested strains) showed chitinase activity.

The successful symbiotic relationship between endophytic bacteria and their hosts could be attributed to hydrolytic enzymes that assist endophytes to permeate plant tissues. Hydrolytic enzymes secreted by endophytes may promote plant growth through hydrolysis of the phytopathogen cell wall [48]. Catalase enzymes are the first defense line in microbes that scavenge toxic free radicals formed due to abiotic and biotic stress and, hence, indirectly promote plant growth [49].

Chitinase activities could promote plant growth through biocontrol of phytopathogenic fungi through hydrolysis of chitin in the fungal cell wall [34]. This phenomenon could explain the efficacy of these endophytic bacteria for the in vitro antagonistic activity of phytopathogenic fungi. In the same line, Hallmann et al. [50] reported that the extracellular enzymatic activities of endophytic microbes improved the induced systematic resistance in the plant. The hydrolytic enzymatic activities of these endophytic bacteria could increase the degradation of protein and polysaccharides, and thus can be useful in industrial sectors [51].

Another strategy used via endophytic bacteria to promote plant growth is ammonia production and solubilization of phosphates [52]. Endophytic bacteria that produce beneficial metabolites for the plant, including ammonia, can prolong the root and shoots of the plant, as well as increase the fresh weight of the inoculated plant [53]. Interestingly, all tested bacterial species in this study had the capacity to producing ammonia with varying degrees. We propose that bacterial endophytes could produce ammonia from chains of amino acids through proteolytic activity where ammonia is the result of the hydrolysis of amide nitrogen, or by the initial proteolytic breakdown of the molecule followed by deamination, as was reported for *Corynebacterium* [54].

Microorganisms also produce ammonia from the hydrolysis of urea into ammonia and carbon dioxide. Ammonia can help meet the nitrogenous needs of the plant and also helps the plant to minimize colonization by plant pathogens [55]. The obtained data are consistent with AlKahtani et al. [8], who reported that the endophytic bacteria obtained from medicinal plants, collected from Saint Katherine Protectorate, South Sinai, Egypt, produced ammonia, which was indicated in culture media by adding Nessler’s reagent. Phosphorus is the major nutrient mandated for plant growth with a high amount. However, phosphate solubilization is required to convert insoluble forms to available ones that can be utilized by plants.

These conversions can be accomplished using different rhizospheric and endophytic microbes [56]. Rhizobacteria that have spent their whole life cycle within plant roots do not show an adverse effect on their host or any outside contamination and develop in close association with intracellular plant growth-promoting rhizobacteria or endophytes [57]. Phosphate solubilization by endophytic and rhizosphere bacteria occurs in the rhizosphere and may have a role in plant needs of phosphate [56].

Therefore, we expect that studying the phosphate-solubilizing endophytes could benefit plants as these endophytes may colonize plant roots and aid in the mobilization of soil non-soluble phosphate. In this study, out of seven tested endophytic bacteria, four strains (57.2%) have the potentiality to solubilize tri-calcium phosphate, forming a clear zone on pikoviskaya agar media ranging between 5.6 ± 0.4 and 9.8 ± 0.8 mm. Endophytic bacteria *Bacillus* spp. PI-8 and PI-10 possess the highest efficacy in ammonia production and the solubilization of phosphate. Therefore, these bacterial endophytes can be used to enhance plant growth and decrease the addition of chemical fertilizers, as shown in greenhouse experiments.

Another vital trait assessed in the present study was the antagonistic activity of isolated bacterial endophytes against three different plant pathogenic fungi. All tested bacterial isolates had antifungal activity against *F. oxysporum, A. alternata,* and *P. ultimum* with varied inhibition percentages (Table 4). This phenomenon could be related to different mechanisms, such as pathogenesis, producing active compounds that inhibit fungal growth, or competition between endophytes and pathogen around nutrients [58]. Fadiji and Babalola [59] reported that the endophytic microbes can protect plants against pathogens by different indirect and direct mechanisms. The direct mechanisms include antipiosis, the secretion of lytic enzymes, the production of different phytohormones, phosphate solubilization, forming siderophores, competitions, and use of aminocyclopropane-1-carboxylate (ACC).

The indirect mechanisms include the enhancement of secondary metabolite secretion via plants, the induction of plant resistance, promotion or enhancement of plant growth, predation, and hyperparasites. In the current study, the tested endophytic bacteria had the potential to perform one or more of the previous mechanisms to improve plant growth and protection. The highest inhibition percentages of phytopathogens were mediated due to treatment with *Bacillus cereus* PI-8 and *Bacillus subtilis* PI-10 recoding percentages of 48.9 ± 0.03% and 52.6 ± 0.15% for *F. oxysporum,* 46 ± 0.09% and 50 ± 0.2% for *A. alternata,* and 20 ± 0.2% and 24 ± 0.2% for *P. ultimum*, respectively. According to phytopathogenic data, the obtained endophytic bacterial strains can be used to protect plant against most fungal pathogens.

Notably, *Bacillus* spp. are the most predominant endophytic effective species against plant pathogenic fungi, consistent with many published studies [60,61,62]. This is attributed to the potential of these species to produce diverse active metabolites and different lytic enzymes, such as amylase, protease, and chitinase, which attack the fungal cell wall [62,63]. According to the obtained results, we can conclude that the endophytic strains *Bacillus cereus* PI-8 and *Bacillus subtilis* PI-10 exhibited the highest activities, and therefore it could be used as bioinoculants and to examine their effects on *Zea maize* root growth.

Once bacterial endophytes inhabit plant tissues, a mutualistic relationship between the bacteria and plant is formed. The bacterial endophytes act as producers of biologically active metabolites, while the plant provides the bacterial community with nutrients [19]. In this study, inoculation of the two most potent bacterial endophytes in maize seeds displayed better root length as well as better fresh and dry root weight compared with the controls (un-inoculated seeds). The consistent with Hassan [19], who reported that the inoculation of *Bacillus cereus* Tp.1B and *Bacillus subtilis* Tp.6B in maize seeds showed enhancement in root length and root weight as compared to the controls.

The finding of bacterial endophytes inhabiting medicinal plants and their potentiality as bioinoculants to improve the growth behavior of other plants were recorded by researchers. AlKahtani et al. [8] reported that out of 13 bacterial endophytes inhabiting the leaves of two medicinal plants, *Fagonia mollis* and *Achillea fragrantissima*, the five most potent endophytic strains were utilized as an inoculant to improve the maize plant growth. Zhao et al. [26] showed that the wheat plant growth was improved due to inoculating their seeds with bacterial endophytes isolated from the medicinal plant *Lonicera japonica*. Clearly, the bacterial endophytes obtained in the current study possess various plant growth-promoting traits; hence, these bacterial endophytes could be used as bio-inoculants to improve and enhance the growth performance of other plants.

Bacterial endophytes can influence plant growth through the availability of nutrients, which has positive impacts on plant growth traits [8]. In this study, two endophytic bacterial strains and their consortium showed significant improvement in the plant heights and the fresh and dry weight of shoot and roots compared to un-inoculated plants (control). Endophytic microbes that produce phytohormones, e.g., IAA, demonstrated enhanced shoot and root lengths, as well as improved the number of root tips that improved the nutrient uptake, hence improving the plant growth traits [64]. Phosphorus (P) is one of the important macronutrients for plants but is unavailable because it is found in an immobilized form.

Microorganisms, particularly endophytic bacteria isolated from different plants, have the ability to phosphate solubilize [65]. Dias et al. [66] showed that *Bacillus subtilis* and *Bacillus megaterium*, two endophytic bacteria obtained from strawberries, exhibit high phosphate solubilization and then enhance plant growth. Potassium (K) is another important element for metabolic responses because it acts as an activator for different enzymes [67]. N-fixing endophytic bacterial species are considered an eco-friendly approach to increase the N content and, hence, improve plant growth. In this study, *Zea mays* L. plants inoculated with two endophytic bacteria either individually or in consortium enhanced their P content compared with the control plants.

Endophytic bacterial inoculation increased N and K ions, however, this was not significant compared with the controls. The availability of nutrients such as P, N, and K, due to inoculation by plant growth-promoting bacteria, can be attributed to the efficacy of the synthesis of organic acids that increase nutrient uptake [68]. The enhanced nutrient uptake can be attributed to the efficacy of bacterial endophytes to synthesize growth regulators at the root interface, which improves root development and increases the adsorption of nutrients and water from the soil [69].

## 4. Materials and Methods

### 4.1. Material Used

All reagents and medium components used in this study were analytical grade and obtained from Sigma Aldrich. Moreover, all reactions were achieved using distilled water (dis. H_2_O). The Kits and universal primers used in molecular identification were obtained from QIAGEN. *Zea mays* L. seeds (Cultivar Giza 9) were obtained from the Agricultural Research Center, Field Crop Research Institute, (FCRI), Giza, Egypt. The soil used for the greenhouse experiment was obtained from the Faculty of Agriculture, Al-Azhar University, Cairo, Egypt.

### 4.2. Sample Collection

**Figure 7:**
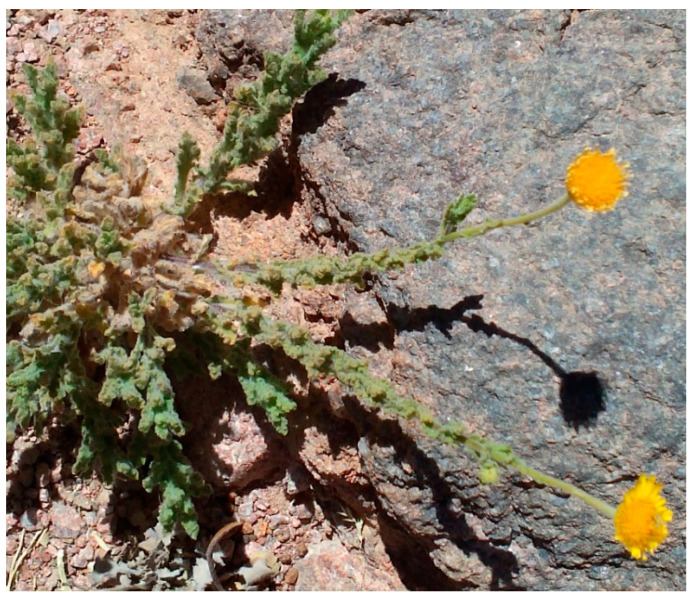
*Pulicaria incisa* (Lam.) DC, grown in Wadi al-Zwatin, Saint Katherine, South Sinai, Egypt.

Healthy leaves of *Pulicaria incisa* (Lam.) DC. (family: Asteraceae) were collected from Wadi al-Zwatin (28.53919°N, and 33.92044°E), Saint Katherine, South Sinai, Egypt. The plant samples were kept in sterile polyethylene bags and transported to the laboratory in a cooling chamber (4 °C) and subjected to selective isolation procedures within 24 h of collection. The plant identification was tentatively recorded in the field with help of local floristic workers, and the botanical identification was carried out at the herbarium of Botany and Microbiology Department of Al-Azhar University. The plant picture is shown in Figure 7.

### 4.3. Isolation of Bacterial Endophytes

The collected plant leaves were washed by running tap water to remove adhered epiphytes and soil debris. After drying under sterile conditions, the surfaces were sterilized by subsequentially soaking them in the following solutions: sterile distilled H_2_O for 1 min, 70% (*v*/*v*) ethanol for 1 min, sodium hypochlorite solution (2.5%) for 4 min, and 70% (*v*/*v*) ethanol for 30 s, and finally the surface-sterilized tissues were washed thrice with sterile distilled water. The last washing water was plated onto fungal, bacterial, and actinomycete culture media of Czapek Dox agar, nutrient agar (NA), and starch nitrate agar, respectively. The surface sterilization was confirmed by the absence of any microbial growth onto the previous cultural media [46,70].

The sterilized leaf samples were aseptically cut into 6 mm fragments and plated onto NA media supplemented with nystatin (25 µg/mL) to suppress fungal growth and were incubated at 37 °C for 48 h. Colonies around the plant segments were purified and preserved in NA slant for further study.

### 4.4. Identification of Endophytic Isolates

The endophytic bacterial isolates obtained from the surface-sterilized leaves were subjected to morphological, physiological, and biochemical tests for preliminary identification on the basis of standard keys of Identifications Bergey’s Manual of Systematic Bacteriology [71,72].

Molecular identification was carried out on the basis of amplification and sequencing of bacterial 16S rRNA gene, where genomic DNA was extracted according to the modified method [73] and the PCR protocol was achieved according to [8]. Briefly, individual colonies from bacterial growth plate were picked up using a sterile toothpick and resuspended in 50 μL of sterile deionized H_2_O. The cell suspension was set in a water bath at 97 °C and heated for 10 min, the cell lysate was forced (15,000× *g*, 10 min), and the upper layer containing the DNA was recovered. The content of extracted DNA was calculated by detecting its absorbance at 260 nm using a UV spectrophotometer. A fragment of 16S rDNA was PCR-amplified using the bacterial universal primers 27f (5′-AGAGTTTGATCCTGGCTCAG-3′) and 1492r (5′-GGTTACCTTGTTACGACTT-3′). The PCR tube contained 1 × PCR buffer, 0.5 mM MgCl_2_, 2.5 U *Taq* DNA polymerase (QIAGEN Inc., Germantown, MD, USA), 0.25 mM dNTP (Deoxynucleoside triphosphate), 0.5 μM primer, and approximately 5 ng of bacterial genomic DNA. 

The cycling of PCR conditions was 94 °C for 3 min, 30 cycles of 94 °C for 0.5 min, 55 °C for 0.5 min, 72 °C for 1 min, and a final extension performed at 72 °C for 10 min. The PCR products were forward- and reverse-sequenced using the Applied Biosystem’s 3730xl DNA Analyzer technology at Sigma company, Cairo, Egypt.

Sequences were then compared with 16S rRNA sequences in the GenBank database using BLAST (Basic Local Alignment Search Tool). The sequences retrieved from this study were deposited in GeneBank under accession numbers from MT994669 to MT994675. Multiple sequence alignment was performed using ClustalX 1.8 software package (http://www-igbmc.u-strasbg.fr/BioInfo/clustalx) and a phylogenetic tree was constructed by the neighbor-joining method using MEGA (Version 6.1) software. The confidence level of each branch (1000 repeats) was tested by bootstrap analysis.

### 4.5. Characterization of Endophytic Bacteria

#### 4.5.1. Salinity Tolerance

The identified endophytic bacterial strains were cultured either with different NaCl concentrations (200, 400, 600, 800, and 1000 mM) or without NaCl to detect sublethal dose for each organism. The results were reported as plus or minus according to bacterial ability to grow in the presence/absence of NaCl [74].

#### 4.5.2. Indole Acetic Acid (IAA) Production by Bacterial Endophytes

The potency of the identified isolated bacterial endophytes for IAA production was quantitatively assessed, where the bacterial strains were inoculated in nutrient broth (NB) media amended with 1.0, 2.0, and 5.0 mg mL^−1^ of L-tryptophan or without tryptophan and incubated at 35 °C ± 2 for 7 days in shaking incubator (150 rpm). A total of 5 mL of each culture was aspirated from the fermentation broth and centrifuged at 6000 rpm for 30 min at 4 °C; 1 mL of the supernatant was mixed with 1 drop of orthophosphoric acid and 2 mL of Salkowski’s reagent (300 mL concentrated sulphuric acid, 500 mL distilled water, 15 mL 0.5 M FeCl_3_) and incubated in the dark for 30 min. Development of pink color indicated IAA production; the optical density was measured at 530 nm using a Spectrophotometer (Jenway 6305 UV spectrophotometer, 230 V/50 Hz, Staffordshire, UK). The amount of IAA produced was estimated by a standard IAA graph [75].

According to qualitative screening, we selected the best tryptophan concentration to assess the efficacy of endophytic bacteria quantitatively at different interval times from 2 to 14 days. Samples were withdrawn and centrifuged at 6000 rpm for 30 min. The IAA production was estimated as aforementioned. The experiment for the assessment of IAA production was performed in triplicate.

#### 4.5.3. Screening the Extracellular Enzymatic Activities of Bacterial Endophytes

The production of extracellular hydrolytic enzymes by bacterial endophytes was investigated using the agar diffusion assay method. The different extracellular enzymes including amylase, protease, xylanase, cellulase, chitinase, and catalase enzymes were assessed. A mineral salt agar media (MSA; containing g/L: NaNO_3_, 5; KH_2_PO_4_, 1; K_2_HPO_4_, 2; MgSO_4_·7H_2_O, 0.5; KCl, 0.1; CaCl_2_, 0.01; FeSO_4_·7H_2_O, 0.02; agar, 15; distilledH_2_O, 1L) supplemented with specific substrates was inoculated by endophytic bacteria as spot-dot inoculation. After that, the inoculated plates were incubated for 48 h at 35 ± 2 °C and flooded with specific reagents after incubation periods to visualize halos around the bacterial growth. All experiments were performed in triplicate.

To detect amylase production, we grew the endophytic isolates on MSA supplemented with 1% (*w*/*v*) soluble starch. After the incubation period, the inoculated plates were flooded with 1% iodine. The appearance of clear zones around bacterial growth was measured (mm) to determine the amylolytic activity.

Proteolytic activity of endophytic isolates was assessed through inoculating bacteria on MSA containing 1% (*w*/*v*) gelatin. The hydrolysis of gelatin was observed as a clear zoon around the bacterial colonies after flooding the plates with acidic mercuric chloride as an indicator.

The xylanolytic activity was estimated on MSA supplemented with 1% (w/v) xylan of corn cobs. The efficacy of bacterial endophytes to produce xylanase enzyme was confirmed as a clear zone (mm) appearing around the bacterial growth after flooding the inoculated plates with absolute ethyl alcohol.

The efficacy of endophytic bacteria to produce cellulase enzyme was investigated after inoculation of bacteria on MSA containing 1% (*w*/*v*) carboxymethylcellulose. After the incubation period, cellulase activity was visualized as a clear zone formed after flooding the agar plates with Logule’s iodine solution [46].

Chitinase activity was measured after inoculation of endophytic bacteria on MSA supplemented with 1% of colloidal chitin formed using commercial chitin according to [76]. The efficacy of bacteria to produce chitinase was investigated by measuring the clear zone, which indicated chitin degradation [34].

For catalase activity, a drop or two of hydrogen peroxide (3%) was added to an endophytic isolated colony and observed for the formation of oxygen. Vigorous bubbling indicated a strong catalase reaction.

#### 4.5.4. Ammonia Production

Ammonia production was analyzed using the qualitative method of [77]. Bacterial isolates were tested for the production of ammonia in peptone water. Freshly grown cultures were inoculated in 10 mL peptone water and incubated for 72 h at 35 °C. Nessler’s reagent (1 mL) was added to each tube as a colorimetric reagent for ammonia production. The color change to faint yellow indicated the minimum ammonia production, while the color change from deep yellow to a brownish color indicated the maximum ammonia production.

#### 4.5.5. Phosphate Solubilization Activity

Bacterial endophytic isolates were screened for phosphate solubilization by the procedure of Jasim et al. [78] using Pikovskaya medium (glucose, 10.0 g L^−1^; Ca_3_ (PO_4_)_2_, 5.0 g L^−1^; (NH_4_)_2_So_4_, 0.5 g L^−1^; NaCl, 0.2 g L^−1^; MgSO_4_·7H_2_O, 0.1 g L^−1^; KCl, 0.2 g L^−1^; FeSO_4_·7H_2_O, 0.002 g L^−1^; yeast extract, 0.5 g L^−1^; MnSO_4_, 0.002 g L^−1^; agar, 20 g L^−1^) and bromophenol blue as an indicator. After 3 days of incubation at 35 °C, strains that induced a clear zone around the colonies were considered as positive.

#### 4.5.6. In Vitro Antagonistic Bioassay

The antagonistic activity of identified bacterial endophytes was evaluated against widely prevailing fungal plant pathogens by dual-culture in vitro assay according to [79]. Three fungal plant pathogens represented as *Fusarium oxysporum*, *Alternaria alternata*, and *Pythium ultimum* were obtained from the Plant Pathology Department, Faculty of Agriculture, Zagazig University. Briefly, each endophytic bacterial isolate was spotted at 3 equidistant points along the perimeter of the potato dextrose agar (PDA) plate (3 plates per isolate). After 24 h of incubation at 28 °C in the dark, a 5 mm plug from the leading edge of a 7-day-old culture of each plant pathogenic fungus on PDA was placed in the center of the plate. Plates without bacteria were used as the control. Plates were incubated at 28 °C for 5 days, after which the relative growth inhibition percent was calculated according to the following equation:

$$\mathrm{Growth}\;\mathrm{inhibition}\;\left(\%\right)=\;\frac{R2-R1}{R1}\;\times 100$$

where R1 represents the lengths of radial growth toward the bacteria and R2 represents the lengths of hyphal growth on a control plate.

### 4.6. Effect of the Most Potent Endophytic Bacteria on Root Length of Zea mays Plant

#### Gnotobiotic Root Elongation Assay

The two most potent bacterial isolates PI-8 and PI-10 were selected for their better activities in order to test the effect of their inoculation on root growth performance of maize plants. *Zea mays* L seeds were surface sterilized by soaking in 2.5% sodium hypochlorite for 5 min and 70% ethanol for 1 min, and were then washed by sterile distilled water 5 times. Bacterial pure cultures of PI-8 and PI-10 were grown individually in nutrient broth at 35 ± 2 °C on a shaker at 180 rpm. Bacterial cultures were centrifuged, and cells were suspended in sterilized saline solution to a final concentration of 10^6^ CFU mL^−1^ (Cell Forming Unit mL^−1^) [80]. Pre-germinated and surface-disinfected seeds were incubated with bacterial suspensions at room temperature for 6 h; the broth without microbial inoculation was used to treat control seeds. Control and treated seeds were placed in sterilized glass Petri dishes (ø = 15 cm) with wet sterilized filter papers and incubated at room temperature for 8 days in the dark to measure the root length and biomass. A total of 10 mL of sterilized distilled water was added to each plate as needed. The experimental treatments were conducted in 5 replications, in which there were 6 seeds per each replicated unit. Seed germination was checked every 24 h for 8 days, and root length, as well as fresh and dry weights, were measured at the end of the experiment.

### 4.7. Greenhouse Experiment

#### 4.7.1. Experimental Design and Soil Analysis

A completely randomized pot experiment with 5 replicates for each treatment was performed to investigate the efficacy of the 2 most potent endophytic bacterial isolates (*Bacillus cereus* PI-8 and *Bacillus subtilis* PI-10) individually or in a consortium on the growth performance of *Zea mays* L. as a model plant. The soil used in the greenhouse experiment was classified as sandy soil containing sand, silt, and clay of 96.25%, 1.50%, and 1.25%, respectively. The chemical characters of soil used, including P, K, Na, Ca, and Cl, were present with values 23.5, 15.75, 184.45, 25.50, and 130.25 mg Kg^−1^, respectively.

#### 4.7.2. Bacterial Inoculations

The two most potent endophytic bacterial isolates were inoculated in nutrient broth media (contain g L^−1^: peptone, 5.0; beef extract, 3.0; NaCl, 5.0) for 24 h at 35 ± 2 °C and shaking condition 150 rpm. Seeds of *Zea mays* L. plant (Cultivar Giza 9) were surface-sterilized as follows. Seeds were soaked in sodium hypochlorite (2.5%) for 3 min and washed thrice with sterilized distilled water. The sterilized seeds were left to pre-germinate, and after that, 4 groups of similar germination seeds were separated and 3 of them were soaked in 100 mL of bacterial inoculated culture (adjusted the optical density (O.D.) at 1.0) individually and in a consortium, while the fourth group was soaked in un-inoculated culture (control). All soaked seeds were incubated at 35 ± 2 °C for 4 h on shaking condition (150 rpm). After incubation periods, the soaked seeds were sown in plastic pots filled with 1000× *g* of sand soil. Each plastic pot received 3 germinated seeds, and the plant was grown in a greenhouse at 25–30 °C and irrigated with tap water as required without adding any fertilizers.

#### 4.7.3. Plant Sample Analysis

Plant samples were collected after 27 days of sowing. The following vegetative growth parameters were recorded after separate root and shoot system: plant heights were measured, fresh weight of shoot and root were measured, and dry weight of shoot and root were also measured after placing them in an oven at 70 °C until the weight was constant. Phosphorus, nitrogen, and potassium contents were determined according to the methods described by AOAC International [81].

### 4.8. Statistical Analysis

All results presented are the means of 3 independent replicates. Data were subjected to statistical analysis by a statistical package SPSS v17. The mean difference comparison between the treatments was analyzed by analysis of variance (ANOVA) and subsequently by Tukey’s HSD (honestly significant difference) test at *p* < 0.05.

## 5. Conclusions

The present study showed that the medicinal plant of *P. incisa*, which naturally inhabits arid conditions, is an ecological niche for diverse putative bacterial endophytes. The isolated bacterial endophytes were related to *Agrobacterium*, *Acinetobacter, Brevibacillus, Bacillus, Paenibacillus,* and *Burkholderia.* These endophytes displayed various direct and indirect mechanisms for plant growth promotion, including IAA production, extracellular hydrolytic enzyme activities, ammonia production, and the solubilization of phosphate, as well as in vitro antagonistic activities against three phytopathogenic fungi.

Therefore, the inoculation of maize roots with endophytic bacterial isolates enhanced plant growth and improved biomass production compared to uninoculated plants. This study provides evidence of the potentiality of these endophytes to improve plant production and plant health, leading to improved soil quality and fertility in agricultural sectors. Moreover, these endophytic bacteria inoculated into *Zea mays* plants under greenhouse experiments and improved plant growth compared to un-inoculated plants.

## Figures and Tables

**Table 1:** The 16S rRNA sequence identification of endophytic bacterial strains isolated from *Pulicaria incisa* and their tolerance to salt stress.

Bacterial Strain Code/Accession Number	Homologue Sequences (Sequence Identity %)	NCBI Accession Numbers	NaCl Tolerance			
			100 mM	150 mM	200 mM	250 mM
PI-1/MT994669	*Agrobacterium fabrum* (99.8%)	NR_074266	++	++	+	−
PI-3/MT994670	*Acinetobacter radiresistens* (99.8%)	NR_114074	++	+	−	−
PI-5/MT994671	*Brevibacillus brevis* (99%)	NR_115589	++	++	+	−
PI-8/MT994672	*Bacillus cereus* (99.6%)	NR_115714	++	++	+	−
PI-10/MT994673	*Bacillus subtilis* (99.4%)	NR_113265	++	++	+	−
PI-12/MT994674	*Paenibacillus barengoltzii* (99.3%)	NR_113988	++	+	+	−
PI-15/MT994675	*Burkholderia cepacia* (99.7%)	NR_113645	++	+	−	−

−, +, and ++ refer to no growth, weak growth, and normal growth, respectively.

**Table 2:** Extracellular enzymatic activities of bacterial endophytes isolated from *Pulicaria incisa*.

Bacterial Isolates	The Diameter of Clear Zones (mm)					
	Amylase	Protease	Xylanase	Cellulase	Chitinase	Catalase
PI-1	0 ^b^	14.9 ± 0.1 ^b^	14 ± 0.1 ^ab^	14.7 ± 0.1 ^d^	13 ± 0.2 ^ab^	+
PI-3	0 ^b^	13.5 ± 0.2 ^b^	12 ± 0.2 ^bc^	15.5 ± 0.03 ^cd^	12 ± 0.1 ^b^	+
PI-5	10 ± 0.1 ^a^	14 ± 0.1 ^b^	11.6 ± 0.1 ^c^	15.9 ± 0.1 ^cd^	0 ^c^	+
PI-8	12.8 ± 0.2 ^a^	17.5 ± 0.3 ^a^	16 ± 0.1 ^a^	19 ± 0.1 ^ab^	15 ± 0.2 ^a^	+
PI-10	11 ± 0.2 ^a^	15.6 ± 0.3 ^b^	15 ± 0.2 ^a^	21.8 ± 0.1 ^a^	13.9 ± 0.1 ^ab^	+
PI-12	10.5 ± 0.3 ^a^	14 ± 0.2 ^b^	12 ± 0.2 ^bc^	18 ± 0.1 ^b^	11.5 ± 0.1 ^b^	+
PI-15	0 ^b^	0 ^c^	11 ± 0.2 ^c^	17 ± 0.1 ^bc^	0 ^c^	+

Values within the same column with different letters are significantly different (*p* ≤ 0.05), values are means ± SD (*n* = 3). + meaning the production of bubbles due to catalase enzyme activity.

**Table 3:** Ammonia production and phosphate solubilization of endophytic bacterial isolated from *Pulicaria incise.*

Bacterial Isolate	Ammonia Production	P Solubilization Diameter of the Clear Zone (mm)
PI-1	+	5.6 ± 0.4 ^c^
PI-3	+	6 ± 0.5 ^bc^
PI-5	++	0 ^d^
PI-8	++	9.8 ± 0.8 ^a^
PI-10	++	7 ± 0.7 ^b^
PI-12	+	0 ^d^
PI-15	+	0 ^d^

+ and ++ denote low and high ammonia production, respectively according to color change after adding Nessler’s reagent. Values within the same column with different letters are significantly different (*p* ≤ 0.05), values are means ± SE (*n* = 3).

**Table 4:** In vitro antagonistic activity of isolated bacterial endophytes isolated from *P. incisa* against *Fusarium oxysporum, Alternaria alternata,* and *Pythium ultimum.*

Bacterial Isolate	Percentage of Growth Inhibition (%)		
	*Fusarium oxysporum*	*Alternaria alternata*	*Pythium ultimum*
PI-1	22 ± 0.3 ^e^	23.8 ± 0.2 ^e^	18.5 ± 0.2 ^cd^
PI-3	23.8 ± 0.2 ^e^	22.5 ± 0.3 ^e^	15.9 ± 0.3 ^e^
PI-5	45.6 ± 0.3 ^c^	38.8 ± 0.2 ^c^	17 ± 0.1 ^de^
PI-8	48.9 ± 0.03 ^b^	46 ± 0.1 ^b^	20 ± 0.2 ^b^
PI-10	52.6 ± 0.2 ^a^	50 ± 0.2 ^a^	24 ± 0.2 ^a^
PI-12	24 ± 0.2 ^e^	31.7 ± 0.4 ^d^	19 ± 0.1 ^bc^
PI-15	33 ± 0.1 ^d^	37.5 ± 0.3 ^c^	17 ± 0.1 ^de^

Values within the same column with different letters are significantly different (*p* ≤ 0.05), values are means ± SD (*n* = 3).

**Table 5:** Effect of most potent bacterial inoculations on the growth properties of maize roots.

Bacterial Treatments	Root Length (cm)	Root Biomass (mg)	
		Fresh Weigh	Dry Weight
Control	19.5 ± 1.6 ^c^	1529 ± 43.6 ^b^	305.8 ± 16.6 ^c^
PI-8	28 ± 1 ^a^	1804 ± 36 ^a^	505 ± 26 ^a^
PI-10	25 ± 1 ^b^	1766.8 ± 29.9 ^a^	441.7 ± 14 ^b^

Values within the same column with different letters are significantly different (*p* ≤ 0.05), values are means ± SD. Control is non-bacterial inoculated plants.

**Table 6:** Effect of endophytic bacterial inoculations in growth performance and nutrient contents of *Zea mays* L. plant.

Bacterial Treatments	Plant Height (cm)	Growth Performance				Shoot Nutrient Content		
		Fresh Weight (mg)		Dry Weight (mg)		P (%)	K (%)	N (%)
		Shoot	Root	Shoot	Root			
Control	29 ± 0.5 ^d^	515 ± 3.0 ^d^	1512 ± 4 ^d^	69.7 ± 0.6 ^d^	354.6 ± 4.5 ^d^	0.18 ± 0.006 ^b^	2.06 ± 0.06 ^a^	1.4 ± 0.03 ^a^
PI-8	38.9 ± 0.3 ^b^	651.7 ± 3.8 ^b^	1820 ± 5 ^b^	85.7 ± 2.5 ^b^	514 ± 4 ^b^	0.23 ± 0.006 ^ab^	2.3 ± 0.06 ^a^	1.5 ± 0.02 ^a^
PI-10	34 ± 0.8 ^c^	601.7 ± 4 ^c^	1784 ± 4 ^c^	76 ± 2.6 ^c^	449 ± 1 ^c^	0.2 ± 0.006 ^ab^	2.2 ± 0.03 ^a^	1.5 ± 0.01 ^a^
Consortium (PI-8 + PI-10)	44.9 ± 0.2 ^a^	718 ± 3.5 ^a^	1918 ± 5.5 ^a^	96 ± 1.7 ^a^	596 ± 5 ^a^	0.34 ± 0.01 ^b^	2.4 ± 0.05 ^a^	1.6 ± 0.02 ^a^

Different letters in each column denote that mean values are significantly different (*p* ≤ 0.05), means ± SE (*n* = 3).

## Data Availability

The data presented in this study are available on request from the corresponding author.
